# Unpicking the Emperor’s New Clothes: Perceived Attributes of the Captain in Sports Teams

**DOI:** 10.3389/fpsyg.2019.02212

**Published:** 2019-10-04

**Authors:** Katrien Fransen, Stewart T. Cotterill, Gert Vande Broek, Filip Boen

**Affiliations:** ^1^Department of Movement Sciences, KU Leuven, Leuven, Belgium; ^2^School for Psychology, Sport and Physical Activity, AECC University College, Bournemouth, United Kingdom

**Keywords:** athlete leadership, peer leadership, leader, selection, shared leadership

## Abstract

Much importance has been assigned to the role of the team captain. In this article, we test whether today’s team captains live up to these high expectations. Furthermore, we provide greater insight into the selection procedures leading to a captain’s appointment and assess how this process impacts upon the captain’s perceived leadership qualities. Adopting a mixed methods design, a total of 398 participants (226 players and 172 coaches) listed the attributes of both their current team captain and their ideal captain. Altogether, participants listed 635 attributes for their current team captain and 919 attributes for their ideal team captain. Both inductive and deductive approaches were used to analyze these qualitative data. Furthermore, quantitative data were obtained on the perceived influencers in the captain’s selection process. The results indicated that, although players and coaches expect their team captains to have good motivational and social leadership skills, the selection process is often underpinned by non-leadership factors, such as experience, sport-specific competence, or irrelevant attributes, such as being the daughter of the club president. This discrepancy held for both coaches’ and players’ perspectives, for male and female teams, across sports, and across competition levels. Although coaches were identified as main influencers in the selection process, giving players the deciding vote did not result in captains with better perceived leadership skills. The significant gap between participants’ expectations of the captain and reality highlights the need for implementing a structure of shared leadership. Furthermore, evidence-based leadership development programs are needed to maximize the team’s leadership potential.

## Introduction

Once upon a time there was an emperor so fond of new clothes that he spent all his money on being well-dressed. One day, two weavers knocked on his door with the promise that they could weave the most magnificent fabrics imaginable, with the extraordinary characteristic of becoming invisible to anyone who was unusually stupid. The emperor was very keen to distinguish the wise men and the fools and paid the weavers a lot of money to start their work. Money that would go directly into the weavers’ pockets as not a penny was spent on the looms. Refusing to admit that they were stupid, the emperor himself, the ministers, and the whole town admired and complimented the design and the colors of the clothes. While the emperor was parading naked on his carriage, it was a little boy who revealed the truth: “But the emperor hasn’t got anything on.” The little voice of innocence spread quickly amongst the crowd and caused a sigh of relief as the truth was revealed. The emperor shivered as he realized that the little boy was right and humiliated beat a retreat.

As the tale by Andersen (1837) indicates, sometimes people are afraid to criticize an established view because everyone else seems to think it is good or important. In this article, we focus on one of the emperors of a sports team: the team captain. In analogy with the tale, we will test whether the clothes of the team captain actually symbolize the leadership that they ought to convey or whether, as with the emperor’s clothes, they are “invisible” instead. In other words, we will first investigate whether the attributes of the current captains align with what coaches and players expect from a captain. Second, we will provide more insight in who selected these captains in the first place and whether a different selection process could be more optimal.

Much importance has been assigned to the role of the team captain in sport. For example, according to Mosher (1979), the captain acts as a liaison between the coaching staff and the players, acts as a leader during all team activities both on and off the field, and represents the team at all receptions, meetings, and press conferences. A case study with a volleyball team indicated that the team captain also claimed to play a key role in communicating team-related information and motivating teammates (Filho et al., 2014). Other studies corroborated the high expectations associated with the role of the team captain. For example, Dupuis et al. (2006) emphasized the captain’s role in motivating and supporting teammates and in serving as a communication bridge between players and coaches. Setting the proper example both on and off the field by putting in effort and always working hard were considered as the most important attributes for a team captain. Interviews with American high school sports captains added captain responsibilities such as facilitating relationships within the team, mediating conflicts, enforcing team roles, mentoring young players, and mentally preparing their teammates (Voelker et al., 2011). Cotterill and Cheetham (2017) suggested an even greater load on the captain’s shoulders by identifying the following list of roles: on-pitch decision-maker, motivator, problem-solver, player representative, media liaison, mentor to young players, a player–coach buffer, embodiment of the team’s culture, to challenge the coach, provide feedback on performance, and a number of off-pitch responsibilities. In particular, leading by example was identified as an important attribute.

In other words, coaches, players, spectators, and media all expect a lot from their captains, and these high expectations might put a lot of pressure on the shoulders of the captain (Smith et al., 2018). The question then becomes whether the perceived expectations of leadership match up to the reality of what is provided; just like the emperor and his new clothes.

In their study “The myth of the team captain,” Fransen et al. (2014) hinted that in reality the captains might not meet coaches’ and players’ expectations, suggesting that the clothes of the captains may not be so fashionable after all. Further building on an earlier categorization of Loughead et al. (2006), the authors distinguished between four leadership roles that players can occupy, including two on-field roles (i.e., the task and motivational leader) and two off-field roles (i.e., the social and external leader). The full definitions of these roles can be found in Table 1. According to the assigned responsibilities of the team captain outlined above, one would expect that the captain is perceived as best leader across these four roles (i.e., expected to provide tactical guidance to teammates, to encourage and motivate them, to care for a good atmosphere within the team, and to represent the team to external parties). In contrast with these expectations, a study with 4,451 players and coaches revealed that only in 1% of the teams the captain was perceived as the best leader across the four leadership roles (Fransen et al., 2014). Even more remarkable is that in almost half of the teams (44%), the captain was not perceived as best leader in any of these four roles. Both on and off the field, informal leaders were perceived to be better leaders than the team captain. A more detailed examination revealed that informal leaders outscored the team captain on all leadership characteristics, except for team tenure, with the captains often being the ones playing the longest for the team (Fransen et al., 2018). Although team tenure might be a selection criterion that is regularly used to select team captains, it does not seem to be the most effective strategy with only 1% of the team captains meeting the expectations of players and coaches.

**TABLE 1:** The definition of the four leadership roles, as presented to the participants, based on the research of Fransen et al. (2014).

**Leadership role**	**Definition**
Task leader	A task leader is in charge on the field; this person helps his team to focus on the team goals and helps in tactical decision making. Furthermore, the task leader gives his teammates tactical advice during the game and gives them guidance if necessary.
Motivational leader	The motivational leader is the biggest motivator on the field; this person encourages teammates to go to any extreme; this leader also puts fresh heart into players who are discouraged. In short, this leader steers all the emotions on the field in the right direction in order to maximize team performance.
Social leader	The social leader has a leading role off the field; this person promotes good relations within the team and cares about having a good team atmosphere, for example, in the dressing room, on the bus, or during social activity. Furthermore, this leader helps with conflicts between teammates off the field. He is a good listener and is trusted by his teammates.
External leader	The external leader is the link between his team and the people outside the team; this leader is the representative of the team when dealing with the club management. If communication is needed with media or sponsors, this person will take the lead. This leader will also communicate the views of the club management to the team, for example, regarding sponsoring, club events, and contracts.

When reflecting on previous research on the team captain, five specific limitations emerge. To address each of these limitations, we propose five distinct aims that will be examined in this study. As such, the present study aims to provide a more detailed insight into the role of the team captain. Given the exploratory study nature of the study, no *a priori* hypotheses were formulated.

The first limitation of some previous studies (e.g., Fransen et al., 2014) is that they only focused on a selected set of leadership roles. Although captains might not be perceived as the best leaders in these roles, they could have other important attributes that underpin their selection. While Fransen et al. (2018) adopted a more elaborate list of leadership criteria, it was still a predefined list without any possibility for participants to add alternate attributes.

**Aim 1**. To obtain more insight in why the current captains were selected as captains in the first place.

Second, as most previous studies were based on interviews with the team captains themselves (e.g., Dupuis et al., 2006; Voelker et al., 2011; Camiré, 2016; Cotterill and Cheetham, 2017), we have a clear insight into how team captains perceive their leadership role and associated responsibilities. While Bucci et al. (2012) noted that also coaches expect high-quality leadership from their team captain, there is no empirical evidence on the perceptions of players.

**Aim 2.** To obtain more insight in players’ and coaches’ expectations of their captain. As such, we do not only obtain more insight in the clothes of the sports team’s emperors, but we can also determine whether their clothes live up to the expectations of the crowd.

Third, earlier work has often relied on a very specific sample, in terms of the participants (i.e., mostly only team captains), in terms of the sports under investigation (i.e., often a single sport), and in terms of gender (i.e., either male or female captains).

**Aim 3.** In line with earlier suggestions by Dupuis et al. (2006) and by Holmes et al. (2010), we will examine Aim 1 and Aim 2 from the perspective of both players and coaches, for male and female teams, across different sports, and across different competition levels.

Fourth, to our knowledge, there are no studies to date that provide insight in the appointment procedure of the captains. This is particularly concerning as there is evidence suggesting that captains might be selected based on non-leadership criteria including team tenure (Fransen et al., 2018) or playing position (Melnick and Loy, 1996; Fransen et al., 2016a). In other words, we do not know which people have the most influence in this selection process.

**Aim 4.** To identify the main stakeholders in the selection process of the team captain (i.e., coach, players, club management, or fans), in order to better comprehend how this selection procedure is organized. In other words, to unmask the weavers that wove the emperor’s clothes.

Finally, we will examine whether the nature of the appointment procedure (i.e., whether the coach, players, club management, or fans had the main influence in the decision) affected the perceived leadership qualities of the team captain. In other words, we investigate whether the emperor’s clothes would look more fashionable when being woven by others (e.g., when the players select their captain instead of the coach).

**Aim 5.** To determine to what extent the selection procedure (and the nature of its stakeholders) impacts upon the leadership quality of the team captain.

## Materials and Methods

### Design

A mixed methods approach was adopted for the current study. The use of a mixed-methods approach has been highlighted to be a practical response to the varied demands of understanding the dynamic and multifaceted nature of human practices and the social world (McGannon and Schweinbenz, 2011). Building on this perspective, Gibson (2017) highlighted that a pragmatic, problem-focused approach demonstrates that a range of methods (both qualitative and quantitative) can be mixed in a particular study.

### Procedure

We recruited participants by using a database of coaches and players who participated in a previous study (e.g., Fransen et al., 2014). The players and coaches from the previous study who indicated that they wanted to be informed of the results (*N* = 3,938) received a detailed feedback report on completion of the study. In addition to the feedback report, we invited these players and coaches to participate in the current study on leadership and the role of the team captain in their sports. After providing their informed written consent, interested players and coaches were referred to an online survey designed specifically for the purpose of the current study. The research project was approved by the institutional review board of the first author’s university.

### Participants

In total, 398 people participated in our survey, of which 226 were players and 172 were coaches. The rather lower response rate (i.e., 10%) can be explained by the fact that the invitation to participate was attached to a feedback report and therefore not the main topic of the email. Also, no reminder emails were sent. Descriptive statistics of all participants can be found in Table 2. The age of participants ranged between 15 and 51 years for the players and between 22 and 69 years for the coaches. All players and coaches were active in Flanders (Belgium). The range of sports reflects the distribution within the recruitment pool of participants that were involved in the original study (Fransen et al., 2014), in which basketball, volleyball, and soccer were the main represented sports.

**TABLE 2:** Descriptive statistics of the participants.

	**Players (*N* = 226)**	**Coaches (*N* = 172)**
Age	25.74 (*SD* = 7.18)	45.84 (*SD* = 11.58)
Years of experience in the sport	15.06 (*SD* = 6.64)	19.25 (*SD* = 12.32)
Sex	108♂118♀	160♂10♀
Sex of the team	108♂118♀	125♂45♀
**Sport**		
Basketball	81	44
Handball	5	8
Hockey	5	1
Ice hockey	6	
Netball	13	3
Rugby	4	2
Soccer	13	67
Volleyball	93	44
Water polo	6	2
**Competitive level**		
National level	68	52
Provincial level	128	77
Regional level	13	12
Recreational level	10	2
Youth level	7	29

### Measures

#### Attributes of the Current Captain and of the Ideal Captain

For Aim 1, we asked participants an open question, namely for which reasons/attributes/characteristics their current team captain was chosen to be the team captain. For Aim 2, participants were asked an open question to identify the most important attributes of the ideal team captain. This approach was adopted to enable a true perspective regarding the required attributes to emerge, as it was important not to constrain or prejudice the views of the participants by outlining a range of potential options or categories.

#### Team Captain Election

First, participants were asked whether the current team captain had been appointed as captain before they joined the team (and maintained the captain’s position ever since without a new selection) or whether the appointment has occurred while they were part of the team. Second, to obtain more insight in the appointment process of the team captain (i.e., Aim 4), participants of the latter group were asked to what extent the following four stakeholders had an influence on the appointment of the team captain: (1) the coach, (2) the players, (3) the club management, and (4) the fans. Participants rated the influence of each of these stakeholders on a scale from 1 (*no influence at all*) to 5 (*very strong influence*). Furthermore, they had the possibility to identify additional sources of influence.

### Data Analysis

Both inductive and deductive approaches were used to analyze the qualitative data, as recommended by Pope et al. (2000). This combination of both inductive and deductive approaches is considered an optimal strategy in analyzing data as it ensures that the analysis is guided by existing theories as well as by the obtained data (Cresswell and Eklund, 2007; Fletcher and Arnold, 2011). The inductive process began with the identification of analytical categories as they emerged from the data (i.e., developing hypotheses from the research field upward rather than defining them *a priori*; Pope et al., 2000). In other words, the obtained attributes for both the current captain and the ideal captain were read and reread to identify lower-order themes that centered on particular behaviors or characteristics. These lower-order themes were subsequently combined where appropriate to form higher-order themes (Aronson, 1995).

The construction and naming of these higher-order themes was in part informed through deductive content analysis based on the leadership categorization framework, developed by Fransen et al. (2014) (Elo and Kyngäs, 2008). This framework includes four leadership roles that players can occupy in the team; two on-field and two off-field roles (for definitions, see Table 1). Informed by this theoretical framework, four higher-order categories were created (i.e., task, motivational, social, and external leadership). Next, the lower-order themes are reviewed for content and coded for correspondence with or exemplification of the identified categories (based on the definitions, presented in Table 1). Informed by previous theorizing on the particular attributes of each of these four leadership roles, we were able to include additional lower-order themes in the four overarching leadership categories (Fransen et al., 2018).

It is important to emphasize that the key point of our approach was to be inclusive (i.e., a characteristic for an inductive approach). Therefore, two additional categories (including 13 lower-order themes in total) emerged that reflected the breadth of responses and perspectives articulated by the participants in the current study (Pope et al., 2000).

Finally, we performed a frequency analysis to illustrate how often each lower-order theme was mentioned by both coaches and players (Neuendorf, 2002). As Sandelowski (2001) noted, a frequency analysis has the benefit of avoiding the major pitfalls in qualitative analysis, such as overweighting vivid accounts of events, underweighting data that do not conform to the pattern the researcher wants to find, or regressing to the mean in which contradictions or messiness is averaged out.

To ensure the quality of the qualitative data, a non-foundational approach was adopted (Smith and Caddick, 2012). The specific criteria that were adopted for judging the quality of this research included the contribution it makes to the field, its coherence, its sincerity, its resonance, and its credibility (Tracy and Hinrichs, 2017). A key aim of this study was to co-construct knowledge that contributes to the understanding of the participants’ perspective on the role and function of the captain and to understand the nature of the selection process. This was achieved by using specific detailed illustrative quotes. Regarding resonance, the core aim of the process was to produce findings that are valuable in the team sport context (Tracy and Hinrichs, 2017).

In addition, the obtained quantitative data on the influencers in the team captain’s selection process were analyzed by using SPSS (version 24). A Shapiro–Wilk test indicated that the observed distribution of all four variables deviated from the normal distribution (all *p*’s < 0.001). As such, we opted for non-parametrical analyses. More specifically, we used the Related-Samples Friedman’s Two-Way Analysis of Variance by Ranks to compare the perceived influence of the four stakeholders (i.e., coach, players, club management, and fans).

In addition, we used Independent Samples Mann–Whitney *U* tests and Kruskal–Wallis tests to examine the differences with respect to the influence of a particular stakeholder between coaches and players, male and female captains, across different sports, and across different competition levels.

## Results

### Qualitative Themes

**FIGURE 1:**
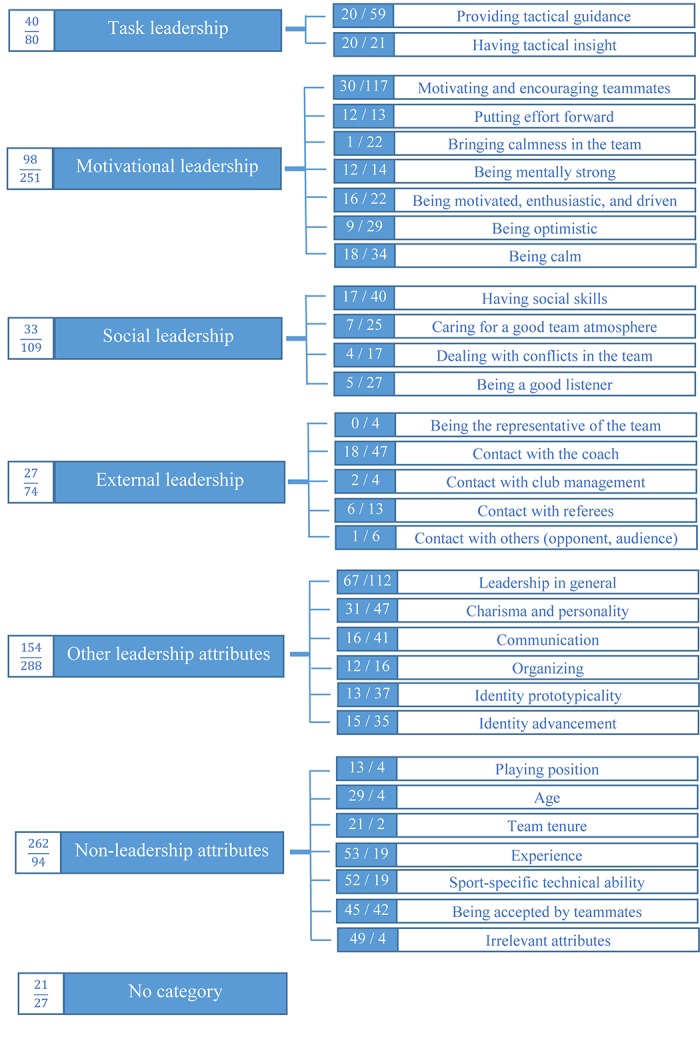
Schematic overview of the higher-order categories (on the left) and the lower-order themes (on the right). The frequencies are presented as “number of citations of current captain/number of citations for ideal captain.

The qualitative data yielded 31 lower-order themes that converged into six higher-order themes. The higher-order themes were: task, motivational, social, and external leadership, other leader attributes, and non-leadership attributes. Figure 1 presents a frequency analysis that illustrates the number of players and coaches that mentioned each theme, either with respect to their current team captain or with respect to their ideal team captain. Table 3 provides more detail by presenting the frequencies cited by coaches and players separately.

**TABLE 3:** Frequency analysis of the characteristic attributes of the current team captain and the ideal team captain, as perceived by players and coaches.

	**Current team captain**			**Ideal team captain**		
		
	**Total**	**Players**	**Coaches**	**Total**	**Players**	**Coaches**
**Task leadership**	40	19	21	80	44	36
Providing tactical guidance to teammates	20	10	10	59	35	24
Having tactical insight	20	9	11	21	9	12
**Motivational leadership**	98	37	61	251	152	99
Motivating and encouraging team members	30	15	15	117	82	35
Putting effort forward	12	4	8	13	4	9
Bringing calmness in the team	1	1	0	22	12	10
Being mentally strong	12	5	7	14	8	6
Being motivated, enthusiastic, and driven	16	5	11	22	11	11
Being optimistic	9	1	8	29	21	8
Being calm	18	6	12	34	14	20
**Social leadership**	33	14	19	109	75	34
Having social skills	17	6	11	40	24	16
Caring for a good team atmosphere	7	3	4	25	20	5
Dealing with conflicts in the team	4	3	1	17	13	4
Being a good listener	5	2	3	27	18	9
**External leadership**	27	12	15	74	29	45
Being the representative of the team	0	0	0	4	4	0
Contact with the coach	18	7	11	47	11	36
Contact with club management	2	2	0	4	4	0
Contact with referees	6	2	4	13	7	6
Contact with others (opponent, audience)	1	1	0	6	3	3
**Other leadership attributes**	154	52	102	288	131	157
Leadership in general	67	28	39	112	52	60
Charisma and personality	31	6	25	47	18	29
Communication	16	6	10	41	17	24
Organizing	12	6	6	16	10	6
Identity prototypicality	13	1	12	37	12	25
Identity advancement	15	5	10	35	22	13
**Non-leadership attributes**	262	150	112	94	47	43
Playing position	13	8	5	4	0	4
Age	29	20	9	4	1	1
Team tenure	21	14	7	2	0	0
Experience	53	33	20	19	9	10
Sport-specific technical ability	52	27	25	19	7	12
Being accepted by teammates	45	14	31	42	28	14
Irrelevant attributes	49	34	15	4	2	2
**No category**	21	10	11	27	18	9

To determine which lower-order themes belonged to one of the four leadership roles, we based ourselves on (1) the definition of the leadership roles (see Table 1; Fransen et al., 2014); and (2) a previous analysis that identified the most important characteristics of each of these leadership roles (Fransen et al., 2018). In addition to these four leadership roles, we identified two other categories; (1) other leadership attributes that could not be included in one of the four leadership roles; and (2) non-leadership attributes. The “other leadership attributes” category included six lower-order themes. The first theme (*Leadership in general*) involves general leadership attributes, which, although they apply to any of the four leadership roles outlined above, are formulated in such a general way, that we could not include them in any leadership role in particular. The second theme (*Charisma and personality*) involves attributes such as charisma, character, mentality, and presence. The third category (*Communication*) involves attributes such as assertive, outspoken, strong communication skills, and vocal. Although good communication is also characteristic for each of the four leadership roles, it cannot be appointed to one in particular. The same holds for the fourth theme (*Organizing*), which involves attributes such as talent in organizing, taking initiative, and making clear arrangements on and off the field.

The last two lower-order themes are types of identity leadership. The social identity approach to leadership (Haslam et al., 2011) asserts that leaders are able to influence their team members to the extent that they create a shared team identity (i.e., a shared sense of “we” and “us”). Two important dimensions of this identity leadership are identity prototypicality and identity advancement. *Identity prototypicality* (i.e., “Being one of us”) is about representing the unique qualities that define the team and what it means to be a member of the team. Example attributes included in this category are “being a model team member” and “being the largest common denominator of the team” (Steffens et al., 2014). *Identity advancement* (i.e., “Doing it for us”) refers to advancing and promoting the core interests of the team. It is about standing up for and defending the group interests, rather than the personal interests (Steffens et al., 2014). Example attributes included in this category are “being team-minded,” “being a team player,” “putting the team ahead, instead of being an egoist,” and “standing up for the team.”

The fifth category includes attributes that are not directly related to leadership abilities. Some of the lower-order themes can, however, be indirectly related to leadership. For example, extensive experience in the sport and sport-specific technical ability potentially underpin task leadership (Fransen et al., 2018). However, since these attributes do not necessarily point to leadership qualities, we did not add them to the first category. Likewise, also the acceptance by teammates (Fransen et al., 2015b), playing position (Fransen et al., 2016a), age, and team tenure (Loughead et al., 2006) can all be indirectly linked to leadership. However, although good leaders might on average be older, more experienced, better skilled, and play on a central position, none of these attributes guarantee high-quality leadership.

In addition, we assembled all attributes that were considered as irrelevant to leadership. These attributes could not even indirectly be linked with leadership and were mainly mentioned as reasons why the team captains were selected. Examples of this last category are: “daughter of the club president,” “son of the team deputy,” “sibling of the previous team captain,” “main sponsor of the team,” “I have no idea,” “because he was a team captain previous season,” “because the club president obliged that he had to play,” “to motivate a player who demonstrated problematic behavior before,” “with a view point on the future, this person can still be captain for years,” etc.

Finally, there were 21 attributes that could not be included in one of the previous categories. These were characteristics that were not directly associated with leadership, but might not be irrelevant in a leadership position either. Example attributes include “modesty,” “well-mannered,” “diplomat,” “rational,” and “punctual.” In order to avoid any contamination of our results, we decided not to include them in one of the previous categories. Their frequencies are listed in a separate category.

In total, 285 participants (i.e., players and coaches) listed attributes of their current team captain, whereas 369 participants listed attributes of their ideal team captain. This difference in response rate might be related to the fact that the team captain was appointed before players or coaches joined the team and the reasons underlying this selection were not clear to them. Altogether, participants listed 635 different attributes for their current team captain and 919 different attributes for their ideal team captain. This discrepancy suggests that players and coaches expect more than what their team captain has to offer.

### Aim 1 – Qualitative Insight Into the Perceived Attributes of the Current Captain

With respect to the current team captain, Table 3 reveals that team captains are mainly selected because of attributes that are not directly related to leadership (*N* = 262). More specifically, experience and sport-specific technical abilities are cited most often as reasons underpinning the captain’s selection. In other words, both coaches and players agreed that the best players with most experience are usually selected as team captains. These attributes can indirectly be linked to task leadership since, on average, task leaders have more experience and a higher skill level than their peers (Wood and Karten, 1986; Fransen et al., 2018). It is important to keep in mind though that neither experience, nor sport-specific skills, guarantee high-quality leadership.

More remarkably is that often attributes that are not relevant at all (e.g., being the sibling of the previous captain) can also influence the appointment of the captain. These findings suggest that the emperor’s clothes might not be so fashionable after all. A possible conclusion is that actual leadership responsibilities, as previously highlighted in the literature, are just not that frequently used when selecting team captains, which brings us to Aim 2.

### Aim 2 – Qualitative Insight Into the Perceived Attributes of the Ideal Captain

To obtain a better insight in the expectations of coaches and players, we asked them to identify the most important characteristics of their ideal team captain. The results, presented in Table 3, reveal that leadership attributes clearly outweigh the non-leadership attributes when it comes to the ideal captain. Apparently, both players and coaches do expect the captain to have good leadership skills. In particular having motivational leadership qualities (i.e., motivating and encouraging team members) appears to be the most frequently cited attribute of ideal team captains according to both players and coaches (*N* = 251). Also social leadership qualities (i.e., having social skills, caring for a good team atmosphere, and being a good listener) were frequently mentioned as an important attribute for team captains (*N* = 109). While experience and sport-specific abilities (both indirectly related to task leadership) underpin the captain’s selection, the motivational and social attributes are most highly valued in the ideal captain.

Other important attributes for the team captain are the provided task guidance on the field, the contact with the coach, and both identity prototypicality (i.e., being a model team member) and identity advancement (i.e., putting the team ahead), despite the fact that only rarely current team captains are selected based on these leadership skills.

When comparing the frequencies of each of the subcategories, we note that each of the leadership categories was mentioned twice as much for the ideal team captain than for the current team captain. However, while the non-leadership attributes were frequently cited for the current team captain (*N* = 262), they are not considered as important for the ideal team captain (*N* = 94). Only the extent to which the captain is accepted by the teammates remains important. In particular, the contrast for irrelevant attributes is striking (i.e., 49 times cited for current captains compared with only 4 cites for the ideal captain). These findings demonstrate that while leadership skills are considered to be important for team captains, in practice these skills are not always used as selection criteria.

### Aim 3 – Testing the Generalizability Across Male and Female Teams, Sports, and Competition Levels

#### Differences Between Male and Female Teams

To analyze the differences between male and female teams, we should keep in mind that 233 participants were involved in a male team, while only 163 participants were involved in a female team. It is thus logical that the average frequencies in female teams will be smaller (x0.7) than in male teams. The Supplementary Appendix A presents the frequencies of the higher-order categories for male and female teams. The findings reveal that male and female teams use similar criteria to appoint their team captain and both male and female participants had comparable expectations of what a team captain should look like.

#### Differences Across Sports

To compare the differences in frequencies across sports, we only included basketball (*N* = 125), soccer (*N* = 80), and volleyball (*N* = 137), as the other sports counted much less participants (16 or less), and are therefore not representative for the entire sport population. The number of participants should be taken into account when comparing the percentages across sports. The findings presented in the Supplementary Appendix A reveal that when it comes to appointing a team captain, experience is a more important attribute in basketball teams, while sport-specific abilities are more important in soccer and volleyball. Consistent for all the different sports, however, is that being able to motivate and encourage team members is perceived as being of greater value for the ideal team captain. Providing tactical guidance and being accepted by teammates are also valued attributes underpinning the captain’s selection, especially in basketball. In addition, all three sports use attributes that have nothing to do with leadership to make their selection, in particular volleyball teams.

#### Differences Across Competition Level

Since the sample size of some of the competition levels was very low, we distinguished between (1) high level (i.e., national level; 120 participants); (2) low level (i.e., provincial and regional level clustered; 230 participants); and youth level (36 participants). Note that we excluded recreational level from these analyses, as the sample size was not sufficient (i.e., 12) and its recreational nature too different from the other competitive categories to allow clustering. The overall number of participants in each of the remaining categories should be taken into account when comparing the frequencies across levels. The Supplementary Appendix A shows that, on both high and low level, experience and sport-specific abilities are decisive when appointing a captain (besides general leadership qualities), although irrelevant reasons are as likely to underpin the selection process. Also at the youth level, sport-specific abilities are key to the captain’s appointment. In sharp contrast with the attributes of the current captains, the motivational and social leadership qualities are on all levels most frequently mentioned as attributes for the ideal captain.

### Aim 4 – Quantitative Insight in the Captain’s Selection Process

While 94 participants indicated that the team captain was already appointed before they joined the team (69 players; 25 coaches), 286 participants (149 players; 137 coaches) indicated being present when the new team captain was selected. The latter group could thus provide us with more information on the main influencers of this decision.

When comparing participants’ responses on their perceived influence of coaches, players, club management, and fans (presented in Table 4), the Related-Samples Friedman’s Two-Way Analysis of Variance by Ranks revealed a significant difference [χ^2^(3) = 586.50; *p* < 0.001]. Additional pairwise comparisons revealed that the differences between each of the stakeholder’s perceived influence were significant. After adjusting the significance values by the Bonferroni correction for multiple tests, all differences remained significant (*p* < 0.001), with exception of the difference between the perceived influence of fans and club management, which was no longer significant. Although the impact of club management seems small, it should be noted that 18% of the participants indicated that club management did influence the decision process, with 18 participants (5.9%) indicating this influence to be strong to very strong. With respect to the fans, only 15% of the participants ascribed influence to them, with seven participants (2.3%) describing this influence to be strong to very strong.

**TABLE 4:** The means (with standard deviations between parentheses), reflecting the extent to which players, coach, club management, and fans influence the team captain’s selection, according to the perceptions of players and coaches, as well as male and female teams active in different sports on different competition levels.

	**Players**	**Coaches**	**Club** **management**	**Fans**
Total sample	3.11 (1.31)	4.01 (1.23)	1.48 (0.93)	1.23 (0.64)
**Function**				
Players	3.19 (1.32)	3.88 (1.23)	1.58 (1.00)	1.24 (0.65)
Coaches	3.02 (1.28)	4.17 (1.21)	1.37 (0.82)	1.22 (0.62)
**Gender**				
Male teams	3.04 (1.25)	4.06 (1.19)	1.59 (1.03)	1.32 (0.73)
Female teams	3.23 (1.38)	3.94 (1.28)	1.34 (0.74)	1.11 (0.44)
**Sports**				
Basketball	3.12 (1.29)	4.03 (1.19)	1.43 (0.90)	1.17 (0.51)
Handball	3.40 (1.27)	4.20 (1.32)	1.89 (1.05)	1.00 (0.00)
Hockey	4.40 (0.55)	3.80 (1.30)	1.60 (0.89)	1.20 (0.45)
Ice hockey	4.17 (0.98)	3.17 (1.47)	2.50 (1.76)	1.40 (0.89)
Netball	3.00 (1.41)	3.86 (1.29)	1.43 (0.85)	1.07 (0.27)
Rugby	2.75 (1.50)	4.50 (0.58)	1.75 (1.50)	1.00 (00)
Soccer	2.90 (1.20)	4.02 (1.27)	1.53 (0.87)	1.45 (0.85)
Volleyball	3.09 (1.37)	4.05 (1.24)	1.33 (0.79)	1.18 (0.60)
Water polo	3.38 (1.30)	3.75 (1.04)	2.50 (1.41)	1.50 (1.07)
**Competition level**				
Highest level	2.79 (1.31)	4.40 (0.83)	2.21 (1.53)	1.36 (0.93)
National level	3.23 (1.19)	4.05 (1.19)	1.62 (0.88)	1.22 (0.59)
Provincial level	3.08 (1.30)	4.07 (1.21)	1.30 (0.66)	1.23 (0.60)
Regional level	2.89 (1.32)	3.63 (1.38)	1.44 (0.98)	1.22 (0.73)
Recreational level	4.00 (1.41)	2.70 (1.42)	2.10 (1.79)	1.22 (0.67)
Youth level	2.97 (1.49)	4.09 (1.22)	1.48 (1.15)	1.24 (0.75)

In addition, 20 participants listed other people as key influencers. The most frequently cited influencers were the assistant-coach, the team’s representative (i.e., a club member who is responsible for the practical arrangements of the team), or no one since a fixed rule was used to appoint the team captain (in this case being the oldest player).

#### Differences Between Players and Coaches

Related-Samples Friedman’s Two-Way Analysis of Variance by Ranks revealed that both coaches [χ^2^(3) = 283.12; *p* < 0.001] and players [χ^2^(3) = 308.12; *p* < 0.001] perceived significant differences between the perceived impact of the different stakeholders in the captain’s selection process. *Post hoc* pairwise comparisons revealed that the general results reported above held for both coaches and players. Furthermore, independent Samples Mann–Whitney *U* tests revealed no differences between the perceptions of players and coaches with respect to the influence of players, club management, and fans on the selection process. Significant differences between the perceptions of the respondent groups emerged with respect to the influence of the coach; coaches perceived their own impact to be stronger than players did (*U* = 10.18; *p* = 0.01; *r* = 0.14).

#### Differences Between Male and Female Teams

Related-Samples Friedman’s Two-Way Analysis of Variance by Ranks revealed that both male teams [χ^2^(3) = 335.32; *p* < 0.001] and female teams [χ^2^(3) = 250.72; *p* < 0.001] perceived significant differences between the perceived impact of the different stakeholders in the captain’s selection process. Again, *post hoc* pairwise comparisons confirmed previous general findings, with one exception; for female teams, there was no longer a significant difference between the perceived influence of players and coaches, after adjusting the significance level by the Bonferroni correction (*p* = 0.12). Furthermore, Independent Samples Mann–Whitney *U* tests revealed that in both male and female teams, participants perceived the influence of both players and coaches similar. Differences did arise for the influence of club management (i.e., in male teams perceived as slightly stronger than in female teams; *U* = 9.72; *p* < 0.05; *r* = 0.12) and the influence of the fans (i.e., in male teams perceived as stronger than in female teams; *U* = 9.43; *p* < 0.01; *r* = 0.18).

#### Differences Across Sports

When looking more closely at the different sports (Table 4), we observe that in most sports (i.e., 7 out of 9), coaches are perceived to have most influence in appointing the team captain, followed by players, club management, and fans. For our statistical analyses, we only included basketball (*N* = 125), soccer (*N* = 80), and volleyball (*N* = 137), as the other sports counted much less participants (16 or less), and are therefore not representative of the entire sport population.

Related-Samples Friedman’s Two-Way Analysis of Variance by Ranks revealed significant differences between the perceived impact of the different stakeholders in the captain’s selection process in both basketball teams [χ^2^(3) = 190.11; *p* < 0.001], soccer teams [χ^2^(3) = 109.22; *p* < 0.001], and volleyball teams [χ^2^(3) = 215.89; *p* < 0.001]. *Post hoc* pairwise comparisons confirmed that the general findings reported above were valid in each of the three sports, with one exception, namely that basketball players did not perceive the difference between the impact of the coach and that of the players as significant anymore (*p* = 0.08 when adjusting by the Bonferroni correction for multiple tests).

When comparing the strength of influence for each of the stakeholders between the different sports, the independent samples Kruskal–Wallis tests only revealed one difference for the perceived impact of fans [*H*(2) = 7.87; *p* = 0.02]. *Post hoc* tests further clarified that this difference was due to the fact that soccer teams perceived their fans to have more influence in the captain selection process than volleyball teams did (*p* < 0.05, when adjusted with the Bonferroni correction).

#### Differences Across Competitive Levels

Since the limited number of participants in some of the categories, we maintained the same clustering as for Aim 3, and distinguished between (1) high level (i.e., national level; 120 participants); (2) low level (i.e., provincial and regional level clustered; 230 participants); and (3) youth level (36 participants).

Related-Samples Friedman’s Two-Way Analysis of Variance by Ranks revealed that participants from both high competitive level [χ^2^(3) = 177.03; *p* < 0.001], low competitive level [χ^2^(3) = 342.69; *p* < 0.001], and youth teams [χ^2^(3) = 66.66; *p* < 0.001] perceived significant differences between the perceived impact of the different stakeholders in the captain’s selection process. *Post hoc* pairwise comparisons confirmed previous general findings, with one exception; for youth teams, there was no longer a significant difference between the perceived influence of players and coaches, after adjusting the significance level by the Bonferroni correction (*p* = 0.30).

When comparing the strength of influence for each of the stakeholders across the different levels, independent samples Kruskal–Wallis tests only identified significant differences with respect to the perceived influence of the club management [*H*(2) = 15.40; *p* < 0.001]. *Post hoc* pairwise comparisons (with Bonferroni corrections) revealed that this significant difference was due to the fact that at high level, club management was perceived to have a greater influence than at low level (standardized test statistic *Z* = 3.81; *p* < 0.001). Apart from the perceived influence of club management, the perceived impact of the other stakeholders was consistent across competitive levels.

### Aim 5 – Impact of the Selection Procedure on the Captain’s Qualities

In this final section, we test whether the appointment procedure (i.e., whether the coach or the players had the main influence in the decision) affected the perceived leadership qualities of the team captain. Since the influence of the players and the influence of the coach were inversely related (i.e., *r* = −0.27; *p* < 0.001), we created two categories of respondents with respect to the perceived influence in the selection process. The first category (*coach impact*) includes the respondents who indicated that the coaches had strong to very strong influence in the decision process (i.e., scores of 4 and 5), while the players had only low to none influence in the decision process (scores of 1 and 2). The second category (*players impact*) includes respondents who ascribed strong to very strong influence to the players (i.e., scores of 4 and 5) and low to none influence to the coaches (i.e., scores of 1 and 2). The “coach impact category” included 77 participants (i.e., 44 players and 33 coaches), whereas the “players impact category” included 27 participants (i.e., 21 players and 6 coaches). Since the total number of respondents is related with the frequency of the listed attributes, Table 5 includes a scaling factor of 77/27 for the players, in addition to the absolute frequencies.

**TABLE 5:** Frequency analysis of the characteristic attributes of the current team captain if the selection was mainly made by either the coach or the players.

	**Coach as main** **influencers (*N* = 77)**	**Players as main** **influencers (*N* = 27)**	
		
	**Absolute** **frequency**	**Absolute** **frequency**	**Rescaled** **frequency** **(×77/27)**
**Task leadership**	8	2	5.70
Providing tactical guidance to teammates	4	1	2.85
Having tactical insight	4	1	2.85
**Motivational leadership**	26	3	8.55
Motivating and encouraging team members	9	1	2.85
Putting effort forward	6	0	0
Bringing calmness in the team	0	0	0
Being mentally strong	4	0	0
Being motivated, enthusiastic, and driven	3	2	5.70
Being optimistic	1	0	0
Being calm	3	0	0
**Social leadership**	6	2	5.70
Having social skills	2	1	2.85
Caring for a good team atmosphere	2	0	0
Dealing with conflicts in the team	1	0	0
Being a good listener	1	1	2.85
**External leadership**	10	0	0
Being the representative of the team	0	0	0
Contact with coach	7	0	0
Contact with club management	1	0	0
Contact with referees	2	0	0
Contact with others (opponent, audience)	0	0	0
**Other leadership attributes**	30	10	28.52
Leadership in general	11	6	17.11
Charisma and personality	12	3	8.56
Communication	0	0	0
Organizing	2	1	2.85
Identity prototypicality	2	0	0
Identity advancement	3	0	0
**Non-leadership attributes**	65	26	74.15
Playing position	4	0	0
Age	3	6	17.11
Team tenure	3	2	5.70
Experience	13	5	14.26
Sport-specific technical ability	12	5	14.26
Being accepted by teammates	12	2	5.70
Irrelevant attributes	18	6	17.11
**No category**	2	2	5.70

When comparing the absolute frequencies of the coach impact group with the rescaled frequency of the player impact group, some similarities can be noted. Both coaches and players relied mostly on non-leadership attributes, and in particular on the irrelevant attributes, to underpin their captain’s choice. Furthermore, experience and sport-specific technical abilities were for both coaches and players decisive attributes that underpinned their selection.

In addition, some differences between players and coaches emerged. When players had the decisive vote, age was a decisive attribute, while coaches based their selection on the captains’ acceptance by teammates and their charisma (which was not important for players). It is possible, though, that the acceptance by teammates constituted an inherent side effect when players were in charge on who to elect as captain. In other words, when the players are in charge of the decision, the appointed captain would implicitly be accepted by the players and player acceptance might no longer be seen as a formal selection criteria.

Noteworthy is that when coaches were the main influencers in the decision process, the selected team captains were much more likely to be perceived as having motivational leadership skills. Furthermore, coaches attributed much importance to the liaison function of the captain as link between the team and the coach, while this was not a criterion when the players were in charge. The fact that the irrelevant attributes were the most cited attributes in both groups suggests that whether the coach or the players appoint the team captain does not make much difference with respect to the leadership skills of the team captain.

## Discussion

This article aimed to provide a deeper understanding of the role of the team captain by addressing five aims. First, we aimed to identify the perceived characteristics of the team captain. Second, we asked players and coaches what their ideal captain would look like, which allowed us to analyze whether the current captains live up to the expectations of players and coaches. Third, we tested the generalizability of our findings by examining the previous questions from both coaches’ and athletes’ perspective, in male and female teams, across different sports, and across various competition levels. Fourth, we shed more light on the procedures underpinning the captain’s selection by identifying the main influencers of this decision. And fifth, we tested whether the quality of the team captain depended on whether the coach, players, club management, or fans appointed the team captain.

### The Emperor’s Clothes

According to both coaches and players of male and female teams, across sports, and across competitive levels, captains are mainly selected based on attributes that are not directly related with leadership. In total, 262 of such attributes were mentioned by 223 participants (as some participants mentioned multiple lower-order themes that underpinned their selection). At least 78% of the participants thus cited one of these “non-leadership” reasons to underpin, at least partly, their selection.

The most often cited lower-order themes were players’ experience and their sport-specific technical abilities. This finding corroborates previous research demonstrating that skills and ability are indeed characteristic for leaders (Wright and Cote, 2003). Fransen et al. (2018) added that in particular task leaders are characterized by experience and sport-specific technical abilities. While Holmes et al. (2010) found that experience was only an important attribute for male captains, in our study, experience and sport-specific qualities were important assets for both male and female captains. Nevertheless, we should note that in the current study experience was more often mentioned for male captains, while sport-specific technical abilities had the deciding vote when it came to appointing female captains. Although the previously mentioned reasons can indirectly be linked to leadership quality (and to task leadership in particular), it should be emphasized that not all players with experience and sport-specific skills are also suited for the captaincy job.

Noteworthy is that the most often cited reasons (i.e., cited by 17% of all participants) had no link with leadership whatsoever. This can be illustrated by reasons as “daughter of the club president,” “sibling of the previous team captain,” and “to motivate a player who had a history of problematic behavior.” In addition, it should be noted that only 285 participants reported attributes of the current captain, while 369 shared their opinion on the ideal captain. This discrepancy could be caused by the fact that other coaches and players had no idea why their current captain was chosen as a captain. In this regard, the 78% of people who used non-leadership reasons to appoint their captain might even be an understatement.

In addition, previous research tended to focus on the positive leadership characteristics and behaviors of team captains, while neglecting alternative reasons for appointing a team captain that have nothing to do with leadership. Recent studies by, amongst others, Cotterill and Cheetham (2017) and Fransen et al. (2016a) have touched on some factors that are unrelated to leadership (e.g., playing position), but these processes of captain selection need further enquiry. The irrelevant reasons found in our study for appointing the captain were adopted to an equal extent in both male and female teams, across all sports, and across all competitive levels, adding to the scope of this problem. Although sports teams’ emperors in general do have some nice clothes that they can be proud on, it is these latter selection criteria that cause some emperors to parade naked.

### The Clothes of the Ideal Emperor

The next aim of this article was to obtain more insight into what players and coaches expect from their emperor’s clothes. To answer this question, we asked coaches and players for the characteristic attributes of their ideal captain. Our findings indicated that both players and coaches do expect their captain to have good leadership skills. In particular the motivational leadership qualities (i.e., motivating and encouraging team members) were perceived as most important attributes of good team captains. Previous research has indeed often cited motivational skills as an important attribute for team captains (Dupuis et al., 2006; Voelker et al., 2011; Filho et al., 2014). In addition, social leadership qualities were also considered valuable assets for the team captains. These findings contrast previous work indicating that task leadership was perceived as more important than motivational and social leadership (Fransen et al., 2014).

Furthermore, it was important for the ideal captain to be accepted by his/her teammates. This finding corroborates previous work that identified acceptance by teammates as an important attribute for leadership quality (Wright and Cote, 2003; Fransen et al., 2018). Moreover, Fransen et al. (2015b) mapped the entire leadership structure within a team and demonstrated that the extent to which players felt connected with their leader proved to be predictive for players’ perceptions of that leader’s quality. This finding held for each of the four leadership roles (i.e., task, motivational, social, and external leader).

Other frequently cited leadership attributes referred to identity leadership (Haslam et al., 2011). The observed importance of identity prototypicality is in line with previous research emphasizing the importance for a leader to be a role model and to embody the team’s culture (Holmes et al., 2010; Steffens et al., 2014; Cotterill and Cheetham, 2017). Furthermore, it supports the assertion of ice hockey coaches that “fitting within the team’s identity was a requirement for players to wearing the “C” on their jerseys” (Bucci et al., 2012). Moreover, players and coaches perceived their ideal captain as an identity champion, creating a shared sense of “we” and “us” and putting the team’s interest above any personal interests. These findings are in line with previous work that demonstrated that good athlete leaders succeed in creating a shared identity. Moreover, the creation of this shared sense of “us” was the leverage for athlete leaders to impact teammates’ team confidence, their health and well-being, and ultimately their team performance (Fransen et al., 2015a, 2016b, 2019b; Rees et al., 2015).

Our study findings were consistent for both coaches and players of male and female teams, across sports, and across competition levels. This consistency contradicts earlier observed differences between perceptions of players and coaches. For example, Voelker et al. (2011) found that, when judging leadership, coaches focused primarily on athletic ability, while players used a wider range of variables, such as peer acceptance and friendship quality.

Noteworthy in the current study is that the main perceived characteristics underpinning the team captain’s selection (e.g., experience, sport-specific technical abilities, and irrelevant attributes) were not identified as the main characteristics of the ideal team captain. Instead, having motivational and social leadership skills should be listed as key attributes on the desired competence profile of the team captain. These findings suggest that, although coaches and players know what to look for in a captain, they do use other selection criteria when it comes to appointing a team captain.

### Who Is to Blame for Weaving the Emperor’s Clothes?

In this section, we aimed to obtain a better insight in the difference between leadership expectations and the adopted selection criteria used to appoint a team captain. Therefore, we looked more closely into the selection procedure by identifying the main influencers in the captain’s selection process. In both male and female teams, across most sports, and across competition levels, coaches had the most influence in appointing a captain, followed by the players. The predominance of the coach in captain selection has been previously highlighted in a range of studies (Cotterill and Fransen, 2016; Cotterill and Cheetham, 2017), though often the criteria used for selection can be questioned. Club management and fans had significantly lower impact in the selection process. Nevertheless, in, respectively, 18% and 15% of the teams, the votes of management and fans were also important in selecting the captain.

Given that coaches have the deciding vote in most of the teams, it is important to verify whether the captain would have better leadership skills if appointed by the players instead. This was not the case. Both coaches and players used experience, sport-specific technical abilities, and irrelevant attributes as selection criteria for appointing their team captain. Despite these similarities, also some differences can be noted. For example, when coaches appointed the team captain, the captain was perceived as having much better motivational skills than when players had the deciding vote. Furthermore, coaches’ selection was mainly based on acceptance by teammates and the captain’s liaison function between the coach and the player group.

The fact that the irrelevant attributes were the most cited attributes, regardless of whether the coach or the players had appointed the captain, suggests that the leadership quality of the team captain does not depend on who selected the team captain, but rather on the selection criteria used in the process. In other words, whoever wove the clothes of the emperor’s, the clothes did not get any more appealing.

### Practical Implications

The findings from the current study suggest that the selection process of team captains does not always involve leadership criteria, although that is what players and coaches expect from their captain. Although some team captains are selected for their leadership skills, the majority of captains in the current study were selected for reasons such as sport-specific technical abilities and experience, which are not directly related with leadership. Even completely irrelevant reasons regularly underpinned the selection process, regardless of whether the coaches or players had the deciding vote.

While the sport-specific talent and experience dominated the selection process, it is clear that both coaches and team captains value a captain’s motivational and social skills more. The title of captain should thus no longer be an honorary title that is automatically assigned to the best player with most experience (e.g., the player with most caps). Instead, captains should be selected on the basis of their skills to positively impact the group dynamics by motivating their team members and by creating a good team atmosphere.

Furthermore, it should be noted that the expectations of players and coaches encompassed a wide array of different facets, ranging from providing tactical guidance, over motivating, to mediating intra-team conflicts and communicating with club management. In other words, the required expertise of today’s captain encompasses so many different facets, that it exceeds the potential of a single individual. Fransen et al. (2014) indeed revealed that only in 1% of the teams the captain could live up to the expectations of players and coaches and was perceived as best task, motivational, social, and external leader of the team. The question then arises whether these white knights are really indispensable for a good functioning of the team. It seems that the contrary is true. Fransen et al. (2014) revealed that teams in which the four leadership roles (i.e., task, motivational, social, and external leadership) were shared among different persons in the team reported stronger team confidence, higher identification with the team, and were better positioned on the team ranking than teams with one exceptional team captain. Therefore, recent studies have recommended a shared leadership approach, in which a group of leaders, rather than one single captain, take the lead together. Cotterill and Cheetham (2017) highlighted that team captains are often the ones asking for such a leadership group to share the burden of responsibilities.

As such, it might be not the underperformance of the team captain that is the biggest threat for a team’s effectiveness, but rather the discrepancy between what is expected of a captain and what that captain can offer. To illustrate, if players expect their captain to fulfill his leadership responsibilities, they might not be so eager to take up an informal leadership role themselves. Possible reasons for not stepping up might include the fear of stepping on the toes of the captain (as he is after all formally assigned as a leader) or on those of the coach (as the coach might have elected that captain in the first place). In this case, an unexploited potential of the team’s leadership is likely to hinder an optimal performance. Reducing the assigned importance to the role of a team captain, together with implementing a structure of shared leadership by formally appointing different leaders in the team assures that all leaders not only have the leadership talent to fulfill their role but also take their responsibility to step up when necessary (Fransen et al., 2019a).

### Strengths and Limitations

By adopting a mixed-methods design, we obtained rich information with respect to the attributes of both the current and the ideal captain. It is only by moving beyond previous research and combining both viewpoints that we obtain greater insight into how the captain’s leadership lives up to the expectations of players and coaches.

Furthermore, most previous studies on team captains relied on a very specific sample (i.e., either the perception of team captains or coaches; either male or female teams; either elite level or lower level; and mostly only one sport). Our study is the first to investigate the role of the team captain from the perspective of both coaches and players, in both male and female teams, across a variety of sports, and across all the competition levels. In contrast to Holmes et al. (2010), we found high consistency across these different categories.

Although we considered different sports, it should be noted that some of these sports had limited sample sizes. Furthermore, the included sports have a relatively normal captain involvement compared with sports such as rugby union and cricket, where the role of the captain gains importance. For example, Cotterill and Cheetham (2017) highlighted the central role that the captain plays in on-field decision-making in the sport of rugby union. Also, Cotterill (2016) highlighted the fact that in professional cricket teams the captain is not subservient to the coach but occupies a parallel position in the hierarchy of the organization, with a specific responsibility for the strategic focus of the team.

Another limitation of our study pertains to the fact that we only included coaches and players from Belgium in our sample. It is likely that perceptions of leadership in sports differ across cultures, as they do in organizations in general (Dickson et al., 2012). For example, Flemish people are known to be more subservient to appointed leaders than for example the Dutch people, as defined by the power distance index in Hofstede’s taxonomy (Hofstede, 2001). Future research could investigate whether the generalizability of the captain’s attributes across Flanders also holds beyond the borders.

### Interesting Avenues for Future Research

Besides the cultural investigation, some other interesting avenues for future research can be noted. One is to investigate whether the choice of the team captain is related with the leadership style of the coach. For example, a very task-oriented coach might make a good choice when appointing a team captain who provides high-quality motivational and social leadership so that the team can profit from leadership that covers a broader range of leadership aspects.

In addition, the observed results clearly reveal a disparity between why a captain is selected and what players expect from their team captain. In many teams the captain’s selection is not a well-considered process and poor team captain selection practices have been identified as a frequent and critical mistake made by coaches (Gould et al., 2013). Either the captain was already appointed previously and that selection is not reconsidered, or certain fixed rules are adopted to appoint a captain (e.g., oldest player). To ensure that high-quality leadership is provided, a well-thought out process can be recommended in which the coaches and players decide together on what they search for in a captain. Based on these selection criteria, a more well-considered choice can then be made. However, keeping in mind that all the requested qualities often exceed the potential of a single individual, it would be better to work with a leadership team, in which the requested leadership qualities are distributed among different players (Cotterill and Fransen, 2016; Fransen et al., 2017).

The contrast between the high expectations on the one hand and the provided captaincy on the other hand could also be solved by evidence-based leadership development programs. For example, although the identity leadership behaviors (i.e., acting as a role model and doing it for the team) were highly valued attributes for team captains, only rarely did the captains also provide such leadership. This contrast might hint to the fact that captains experience a lack of knowledge on how to implement this behavior in practice. As such, the appointed leaders within the team would clearly benefit from programs aiming to strengthen their leadership qualities by helping them how to develop—that is create, embody, advance, and embed—a collective sense of “us” in their teams.

## Conclusion

Although players and coaches expect their captains to excel in motivational and social leadership, the most often cited reasons for appointing a team captain had no link with leadership whatsoever, thereby suggesting that captains do not live up to the expectations of players and coaches. In other words, although some sports teams’ emperors do wear clothes that hint a leadership attitude, they cannot live up to the crowd’s expectations of what emperors’ clothes should look like. Even worse, the nature of the selection process sometimes even causes proud emperors to parade naked in the streets.

## Data Availability Statement

All datasets generated for this study are included in the manuscript/Supplementary Files.

## Ethics Statement

The research project was approved by the Social and Societal Ethics Committee of the KU Leuven. Participation was voluntary and the APA ethical standards were followed in the conduct of this study.

## Author Contributions

KF led the project. KF and FB contributed to the design of the project. KF collected and analyzed the data, and wrote the manuscript. FB, SC, and GV critically revised the manuscript.

## Conflict of Interest

The authors declare that the research was conducted in the absence of any commercial or financial relationships that could be construed as a potential conflict of interest.
