# Supplementary material for: Unpicking the Emperor’s New Clothes: Perceived Attributes of the Captain in Sports Teams
Source: Front Psychol. 2019 Oct 4;10:2212. doi: 10.3389/fpsyg.2019.02212 (PMC6787266; doi:10.3389/fpsyg.2019.02212)
Supplement: Supplementary file 1 [file Data_Sheet_1.docx]

**Appendix A.** Frequency analysis of the characteristic attributes of the current team captain and the ideal team captain for male and female teams, for the different sports, and for different competition levels.

|  |  | Task leadership | Motivational leadership | Social leadership | External leadership | Other leadership attributes | Non-leadership attributes | No category |
| --- | --- | --- | --- | --- | --- | --- | --- | --- |
| **Gender** |  |  |  |  |  |  |  |  |
| Current team captain | Male teams | 28 | 62 | 20 | 18 | 96 | 142 | 12 |
|  | Female teams | 12 | 36 | 13 | 9 | 57 | 108 | 9 |
| Ideal team captain | Male teams | 44 | 129 | 58 | 45 | 180 | 51 | 17 |
|  | Female teams | 36 | 122 | 50 | 29 | 106 | 39 | 10 |
| **Sports** |  |  |  |  |  |  |  |  |
| Current team captain | Basketball | 13 | 28 | 9 | 11 | 45 | 84 | 7 |
|  | Soccer | 8 | 23 | 6 | 5 | 35 | 37 | 4 |
|  | Volleyball | 10 | 35 | 14 | 9 | 51 | 86 | 6 |
| Ideal team captain | Basketball | 30 | 84 | 36 | 21 | 78 | 30 | 11 |
|  | Soccer | 10 | 50 | 17 | 20 | 69 | 14 | 5 |
|  | Volleyball | 27 | 84 | 39 | 27 | 92 | 31 | 8 |
| **Competition level** |  |  |  |  |  |  |  |  |
| Current team captain | High level | 14 | 24 | 10 | 11 | 56 | 89 | 7 |
|  | Low level | 20 | 61 | 19 | 12 | 71 | 141 | 12 |
|  | Youth level | 5 | 13 | 4 | 4 | 23 | 23 | 1 |
| Ideal team captain | High level | 23 | 81 | 27 | 24 | 114 | 28 | 9 |
|  | Low level | 44 | 146 | 66 | 46 | 130 | 51 | 16 |
|  | Youth level | 11 | 18 | 8 | 4 | 33 | 10 | 1 |
